# Parliament and the Profession

**Published:** 1916-01-22

**Authors:** 


### PARLIAMENT AND THE PROFESSION.

### The Tax on Hospital Spirit.

MR. MCKENNA states that it is proposed that relief should take the form of grants from public funds, and that a Committee is being appointed to advise as to the basis of such grants. We have stated on a previous occasion that it is a misuse of words to describe a rebate of the duty on medicinal spirits as a grant out of public funds.

### The Central Medical War Committee.

IN a lengthy reply to a question, Mr. Tennant stated the duties of the Central Medical War Committee to be: "To organise the medical profession in England, Wales, and Ireland in such a way as will enable the Government to use every medical practitioner fit to serve the country in such a manner as to turn his qualifications to the best possible use; to deal with all questions affecting the medical profession arising in connection with the war; and to report to the Committee of the British Medical Association." As an outcome of conferences between the Committee and the Director-General of Army Medical Services the Committee has been endeavouring to procure and to co-ordinate offers from members of the medical profession of service in the R.A.M.C. Applications for such commissions are decided upon by the War Office after they have been referred to the Committee for consideration in relation to the medical needs of the civil population in the area concerned in each case, and after consultation with the Insurance Commission and, where necessary, with the Local Government Board and the Board of Education.

### Other Matters of Interest.

BADGES FOR THE UNFIT.—Mr. Tennant stated that the question of providing badges for men who have been discharged from the Army as medically unfit is still under consideration.

INOCULATION AND COMPULSORY SERVICE.—Replying to Mr. King, the Under-Secretary for War said he understood it was intended to inoculate with anti-typhoid serum the men who may be compulsorily attested under the operations of the Military Service (No. 2) Bill; in this respect, in fact, they will be in the same position as men who undertake voluntary enlistment.

VACCINATION.—Replying to Mr. P. White, Mr. Hayes Fisher stated that the number of successful vaccinations for which vaccination officers received certificates at all ages in England and Wales were, in 1913, 43,470, and in 1914, 404,616, and the number of declarations of conscientious objection to vaccination in 1913 were 308,235, and in 1914, 312,313.

AUSTRALIAN MEDICAL CORPS.—Captain Amery asked whether Lieutenant-Colonel J. W. Barrett, Australian Medical Corps, who occupied a high position on the staff of the Director of Medical Services, Egypt, was removed from his offices without inquiry as a result of a misunderstanding. Mr. Tennant said he believed that was the case, but a Court of Inquiry was subsequently held in Egypt, and in its finding the Court exonerated Lieutenant-Colonel Barrett, and spoke in the highest terms of his work.

